# Thalidomide enhanced the efficacy of CHOP chemotherapy in the treatment of diffuse large B cell lymphoma: A phase II study

**DOI:** 10.18632/oncotarget.8973

**Published:** 2016-04-25

**Authors:** Dongmei Ji, Qiu Li, Junning Cao, Ye Guo, Fangfang Lv, Xiaojian Liu, Biyun Wang, Leiping Wang, Zhiguo Luo, Jianhua Chang, Xianghua Wu, Xiaonan Hong

**Affiliations:** ^1^ Department of Medical Oncology, Fudan University Shanghai Cancer Center, Department of Oncology, Shanghai Medical College, Fudan University, Shanghai 200032, P.R. China; ^2^ Department of Medical Oncology, West China Hospital of Medicine, Sichuan University, Chengdu 610041, P.R. China

**Keywords:** thalidomide, chop, diffuse large B-cell lymphoma

## Abstract

Cyclophosphamide, doxorubicin, vincristine, and prednisolone plus rituximab (R-CHOP) is the standard treatment for patients with diffuse large B cell lymphoma (DLBCL). However, rituximab cannot be popularly applied in a considerable number of patients with DLBCL because of economic reasons. To develop a new regimen to improve the outcome of these patients is extremely important. In our study, sixty five patients with DLBCL were randomly assigned to thalidomide plus CHOP group (n=32) or to CHOP alone group (n=33). Objective response rates (ORR) and complete remission rates (CRR) were 96.7% and 80.6% in T-CHOP group versus 78.9 % and 57.8 % in CHOP group, respectively (*P* <0.05). At a median follow-up of 96 months, median PFS for T-CHOP group was still not reached yet, and in CHOP group it was 22.9 months (95% CI [0-50.4]). (*P*=0.163). Median overall survival (OS) for T-CHOP group was also not reached, and the estimated median OS for CHOP group was 83.5 months, the difference of OS between the two groups is not significant (p=0.263). But, in patients with Bcl-2 positive and Bcl-6 negative, the median PFS in T-CHOP group was longer than that in CHOP group (111.0 vs 8.5 months (P=0.017). In addition, thalidomide did not significantly increase the grade 3/4 toxicity of CHOP. We concluded that the addition of thalidomide to the CHOP regimen significantly improved the CRR and showed a trend of improving clinical outcome in patients with DLBCL, especially for patients with Bcl-2 positive and Bcl-6 negative B-cell phenotype, without increased toxicity.

## INTRODUCTION

DLBCL is the most common type of malignant lymphoma, accounting for about 31% of all NHL [1]. CHOP scheme has been the standard chemotherapy regimen in the treatment of advanced NHL for many years [2–4]. The five year survival rate of the CHOP regimen in the treatment of DLBCL is about 45%-46% [5]. There are still more than half of DLBCL patients eventually died of the disease.

Rituximab, a chimeric monoclonal antibody targeted CD20 antigen, can be used for the treatment of CD20 positive B cell lymphoma. Compared with CHOP scheme, rituximab plus CHOP (R-CHOP) can significantly improve complete remission rate, and prolong event free survival (EFS) and OS [6, 7]. And FDA has approved this drug for treating chemo-naïve DLBCL. However, according to the Chinese public medical insurance policy, rituximab is not covered by the public medical insurance. Rituximab cannot be popularly applied in a considerable number of patients with DLBCL in China and other developing country. Therefore, it is important to explore a new economic regimen to improve the outcome of this patient population.

Angiogenesis play an important role in tumor invasion and metastasis. Anti-angiogenesis therapy has become an important part of the comprehensive treatment for malignant tumors. It is reported that 60% of the DLBCL specimens showed higher local vascular endothelial growth factor (VEGF) expression, higher VEGF receptor (VEGFR) expression and correspondingly higher micro-vessel density [8], which provides a theoretical foundation for the use of anti-angiogenesis therapy in patients with DLBCL.

DLBCL is a heterogeneous disease with different morphological and molecular characteristics [9]. It can be further divided into two subtypes, germinal center B cells (GCB) subtype and non-GCB subtype [10]. Patients with the non-GCB subtype have a distinctly inferior prognosis, which can be attributed to the constitutive I kappa B kinase (IKK) activity leading to nuclear factor kappa B (NF-κB) activation, but it is not seen in GCB subtype DLBCL [11].

Thalidomide is a kind of glutamate derivatives. In the late of last century, people found that thalidomide can inhibit angiogenesis by blocking bFGF and VEGF, and it can also modulate the immune system by co-stimulating T cell proliferation [12–14]. In recent years, some study found that thalidomide can induce the apoptosis of tumor cells and some found it can improve the weight in cancer patients with cachexia [15, 16]. In addition, thalidomide can also inhibit the IKK activity and block the activation of NF-kappa B [17]. In a phase II study, thalidomide combined with rituximab was applied in refractory or relapsed mantel cell lymphoma patients. This regimen was well tolerated and achieved marked efficacy of 31% complete remission rate (CR) and 81% objective response rate (ORR) [18].

In this open-label, randomized, controlled study, the efficacy and tolerability were compared between thalidomide combined with CHOP (T-CHOP) and CHOP alone in chemo-naïve DLBCL patients. (This clinical trial began in Feb, 2006, and was not registered in ClinicalTrials.gov.)

## RESULTS

### Patients’ characteristics

A total of 66 patients were enrolled between February 2006 and June 2007. One patient assigned to the T-CHOP group did not receive any trial medication because he was found to have pulmonary tuberculosis and he was referred to Shanghai Municipal Center for Disease Control and Prevention (CDC) for further treatment; 65 patients were included in the final analysis (CHOP, n=33; T-CHOP, n=32). One patient in T-CHOP group and two patients in CHOP group had cyclophosphamide and epirubicine dose reduction to 75%. In T-CHOP group, one patient stopped to receive thalidomide after the first cycle of treatment because of the onset of angina, and another three patients continued to receive thalidomide at a dose of 200mg because they had incontinent urinate when the dosage increased to 400mg per day. There were no significant differences between the two groups in any of the baseline clinical characteristics (Table 1). Central review of pathology was performed on lymph node material from 100 % of the patients. All cases entered in the study were included in this report. IPI scores were balanced across groups, with most patients (52.3%) having scores of 0 or 1 (Table 1); although not part of the original inclusion criteria, 43.7% of patients randomized to T-CHOP and 51.5% of those randomized to CHOP had scores of 2 to 5 according to the International Prognostic Index (IPI) [22].

**Table 1:** Clinical characteristics of patients treated with T-CHOP or CHOP

Clinical variable	T- CHOP n(%)	CHOP n (%)	*P* value
Age			0.357
≤60yr	22(68.8)	26(78.8)	
>60yr	10(31.2)	7(21.2)	
Stage			0.390
I or II	17(53.1)	21(63.6)	
III or IV	15(46.9)	12(36.4)	
Serum lactate dehydrogenase			0.897
≤250IU/L	17(53.1)	17(51.5)	
>250IU/L	15(46.9)	16(48.5)	
Performance status			0.137
0 or 1	28(87.5)	24(72.7)	
2	4(12.5)	9(27.3)	
Extranodal involvement			0.492
0 or 1	32(100)	31(93.9)	
≥2	0	2(6.1)	
International prognostic index			0.343
0 or 1	18(56.3)	16(48.5)	
2 or 3	14(43.8)	15(45.5)	
4 or 5	0	2(6.1)	
Median cycles of chemo	6	6	0.16

Abbreviations: T-CHOP, Thalidomide plus cyclophosphamide, doxorubicin, vincristine, and prednisone.

The baseline characteristics were also balanced between CHOP and T-CHOP group in different Bcl-2 and Bcl-6 status patients (see Supplementary Tables 1-4).

### Efficacy

In this study, CR rate in T-CHOP group was higher than that in CHOP group, (80.6% vs 57.8%, P=0.039). Overall response rate (ORR, CR+PR) in CHOP group was 78.8% (26/33) and in T-CHOP group was 96.9% (31/32) (P=0.030, Fisher's exact test, 1-sided) (Table 2).

**Table 2:** Response to treatment with T-CHOP or CHOP

	T-CHOP (n=32) no. (%)	CHOP (n=33) no. (%)	P value
Complete response	26(81.3)	19(57.6)	0.039
Overall response rate	31(96.9)	26(78.8)	0.030
Partial response	5(15.6)	7(21.1)	0.562
Stable disease	1(3.1)	4(12.1)	0.355*
Progressive disease	0	3(9.1)	0.238*

* Fisher's exact test

**Figure 1:**
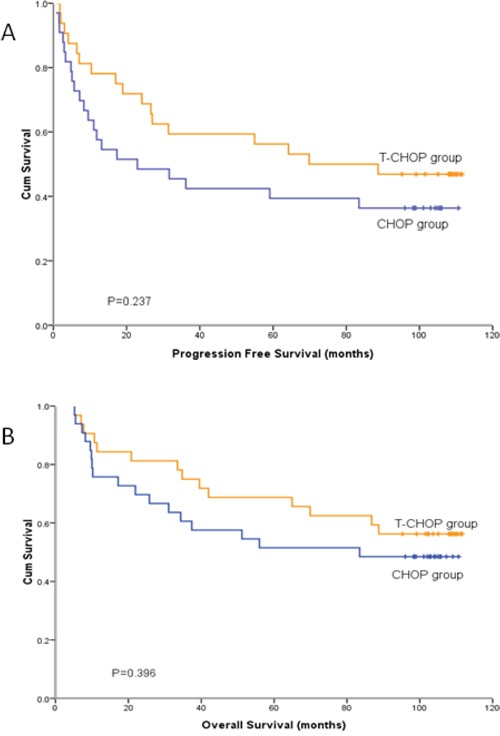
Progression free survival and overall survival curves of 65 patients with diffuse large B-cell lymphoma receiving T-CHOP and CHOP treatment **A.** mPFS were 69.9 months in T-CHOP group and 22.9 months in CHOP group (p=0.237). **B.** mOS were not reach in T-CHOP group and 83.5 months in CHOP group (p=0.396).

**Figure 2:**
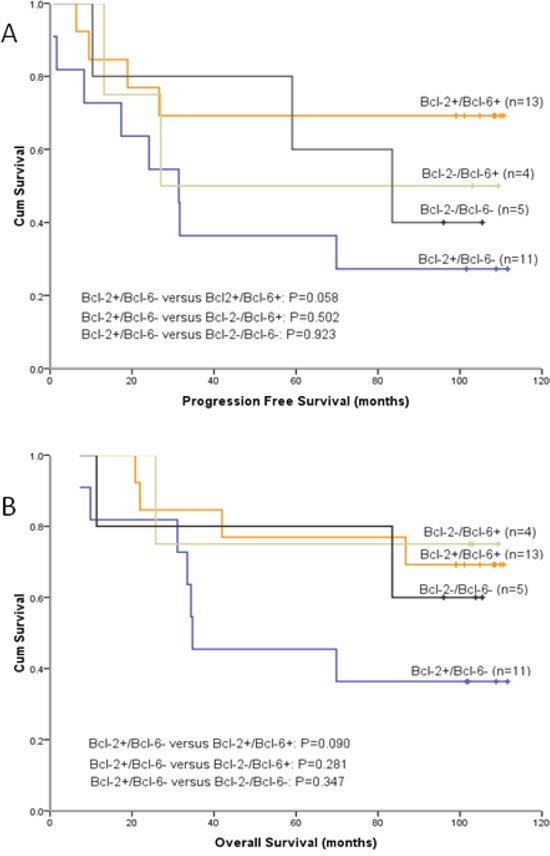
Patients with tumor tissue Bcl-2 positive and Bcl-6 negative (Bcl-2+/Bcl-6-) have the worst outcomes **A.** PFS curves for 33 patients stratified by Bcl-2 and Bcl-6 immunoreactivity. **B.** OS curves for 33 patients in different Bcl-2 and Bcl-6 status.

At a median follow-up of 96 months, median PFS for T-CHOP group was not reached yet (estimated mPFS was 69.9 months), and for CHOP group it was 22.9 months (95% CI [0-50.4]). Median OS for T-CHOP group was not reached, and the estimated median OS for CHOP group was 83.5 months. The survival curves of PFS and OS between these two groups separated, but the differences were not significant, with P values of 0.237 and 0.396, respectively (Figure 1). Figure 2 showed the PFS and OS curves in different Bcl-2 and Bcl-6 status patients. Patients with tumor tissue Bcl-2 positive and Bcl-6 negative (Bcl-2+/Bcl-6-) have the worst outcomes.

**Figure 3:**
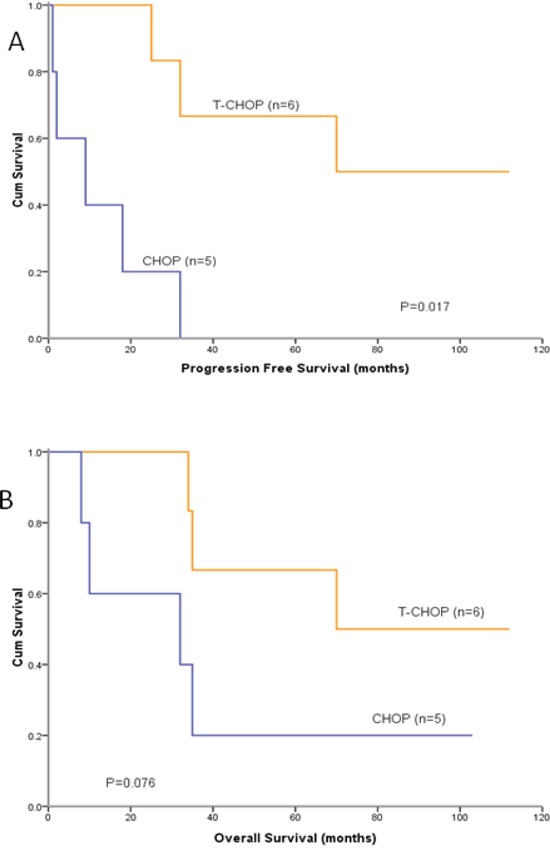
PFS and OS curves in Bcl-2+/Bcl-6- patients treated with T-CHOP or CHOP **A.** PFS in T-CHOP group was significantly longer than that in CHOP group for Bcl-2+/Bcl-6- patients (P=0.017). **B.** Difference in OS between the two subgroups was not significant (p=0.076).

The sample size of Bcl-2+/Bcl-6- patients was 11, and Life Tables method was applied to compare the PFS and OS between T-CHOP and CHOP group. The mPFS for this group of patients treated with T-CHOP was 111.0 months, and for those treated with CHOP was 8.5 months. T-CHOP showed significant superiority over CHOP in PFS in Bcl-2+/Bcl-6- patients (P=0.017) (Figure 3A). In this group of patients, the mOS for T-CHOP group was 111.0 months, and for CHOP group was 31.5 months. The difference of the OS between the two groups was not significant (p=0.076) (Figure 3B).

### Adverse effects

Table 3 presents all reported adverse events in each group. One patient with the history of coronary heart disease in T-CHOP group had chest pain after he received 1 cycle of T-CHOP. Coronary angiography showed a 90% stenosis of the left anterior descending artery. Coronary artery stent was implanted and he continued the chemotherapy of CHOP without thalidomide. More patients in the T-CHOP group than in the CHOP group experienced grade 1/2 adverse events in sinus bradycardia (21.9% vs 3.0%, p=0.027), numbness (59.4% vs 24.2%, p=0.004), somnolence (43.8% vs 0%, p=0.000), and dizziness (40.6% vs 15.2%, p=0.022). It seemed that more patients in T-CHOP had rash (18.8% vs 3.0%, p=0.054). There was no difference in the overall incidence of grade 3 or 4 adverse events between T-CHOP group and CHOP group. None of the patient received prophylactic anticoagulants during the treatment. Deep Venous Embolism (DVT) was not seen in patients treated with T-CHOP and there was no treatment-related death.

**Table 3:** Common toxicities observed in patients in T-CHOP group and CHOP group

	T-CHOP no.(%)	CHOP no.(%)	*P*	T-CHOP no. (%)	CHOP no.(%)	P
	Any grade			Grade 3 or 4		
Neutropenia	25(78.1)	27(81.8)	0.710	10(31.3)	9(27.3)	0.724
Anemia	2(6.3)	6(18.2)	0.258	0	0	
thrombocytopenia	3(9.4)	5(15.2)	0.708	0	0	
Elevated aminotransferase	3(9.4)	7(21.2)	0.303	0	1(3.0)	1.000*
T wave change on ECG	16(50)	3(9.1)	**0.000**	0	0	
Sinus tachycardia	5(15.6)	3(9.1)	0.475*	0	0	
Sinus bradycardia	7(21.9)	1(3.0)	0.027*	1(3.1)	0	0.492*
Vomiting	13(40.6)	8(24.2)	0.158	2(6.2)	1(3.0)	0.613*
Anorexia	8(25.0)	7(21.2)	0.717	1(3.1)	1(3.0)	1.000*
Mucositis	3(9.4)	5(15.2)	0.708*	0	0	
Fatigue	11(34.4)	12(36.4)	0.867	1(3.1)	0	0.492*
Rash	6(18.8)	1(3.0)	0.054*	0	0	
Constipation	18(56.3)	12(36.4)	0.108	1(3.1)	0	0.492*
Acute coronary syndrome	1(3.1)	0	0.492*	1(3.1)	0	0.492*
**Neuro-toxicity**						
*Numbness*	19(59.4)	8(24.2)	**0.004**	2(6.2)	0	0.238*
*Muscular soreness*	3(9.4)	0	0.114*	0	0	
*Tremble*	3(9.4)	0	0.114*	0	0	
*Somnolence*	14(43.8)	0	**0.000**	1(3.1)	0	0.492*
*Dizziness*	13(40.6)	5(15.2)	**0.022**	1(3.1)	0	0.492*
*Incontinent urinate*	3(9.4)	0	0.114*	0	0	

* Fisher's exact test

One patient diagnosed as DLBCL in Dec 2006. He received T-CHOP for 6 cycles and got CR after the first two cycles. This patient relapsed in Apr 2012, he underwent autologous stem cell transplantation and got 2nd CR. In Feb 2016, he had enlargement of the lymphonode. He received biopsy and was diagnosed as mantle cell lymphoma. It's hard to judge whether the MCL is transformed from DLBCL or the secondary malignancy.

## DISCUSSION

In this randomized study, there were three main findings. First, we found that the CRR and ORR in T-CHOP group was significantly higher than that in CHOP group for previously untreated patients with DLBCL (Table 2), which met to the primary endpoint. Second, although the PFS and OS curves in two groups separated apparently since the beginning of the treatment, significantly increased CRR and ORR did not translate into prolonged PFS and OS in this study, which might be mainly attributable to the relatively small sample size of this study. In bcl-2+/bcl-6- subgroup, the median PFS in T-CHOP group was significantly longer than that in CHOP group (Figure 3A). But median OS in T-CHOP group was not significantly longer than that in CHOP group (Figure 3B). Finally, T-CHOP regimen was well tolerated, and produced a pattern of adverse events broadly similar to CHOP alone, with the exception of some mild thalidomide-related reactions such as sinus bradycardia, numbness, somnolence, dizziness and, perhaps, rash. In our study, no DVT was seen in T-CHOP group, which is different from the studies applying thalidomide to patients in west countries [23]. Perhaps the incidence of thalidomide-related thromboembolic events in Chinese patients is not as high as in western country patients [24].

Bcl-2 protein, an anti-apoptotic molecule, is expressed in 22–80% of DLBCLs and Bcl-2 positivity has been reported to be associated with an unfavorable prognosis. Bcl-6 protein is expressed in about 47–84% of DLBCLs. Bcl-6 rearrangement and Bcl-6 protein expression were reported to be associated with favorable clinical outcome [25–28]. In the present study, Bcl-2 positivity and Bcl-6 negativity were found to be parameters predicting a significantly shorter PFS particularly (Figure 2). The Bcl-2 protein is correlated with the NF-kappa B pathway [29], and thalidomide can inhibit the activation of NF-kappa B [17], which might be the possible mechanism of thalidomide can overcome the unfavorable prognosis of Bcl-2. In our study, DLBCL patients with Bcl-2+/Bcl-6- progressed rapidly and had short survival time when they received CHOP regimen, but had relatively longer PFS and OS when received T-CHOP regimen (Figure 3).

Lenalidomide, an expensive analogue of thalidomide, showed significant activity in relapsed DLBCL. In a phase II study, lenalidomide combined with R-CHOP could overcome negative prognostic impact of non–germinal center B-cell phenotype in newly diagnosed DLBCL [29, 30].

Many cost-effectiveness analyses reported that adding rituximab to CHOP in DLBCL patients is cost-effective [31–33]. In 2014, Khor S, et al reported the real world cost and cost-effectiveness of Rituximab for DLBCL patients in Canada. In this report, the addition of Rituximab is associated with improvement in survival but at a higher cost, thus may be not economically attractive in the elderly patients [34]. In china, for a patient with body surface area (BSA) of 1.6 m^2^, about 180,000 RMB Yuan is needed for 6 cycles of Rituximab, about 6,000 Yuan for 6 cycles of CHOP, and about 3,500 Yuan for 6 cycles of thalidomide. In some under-developed areas in middle and west China, many people with CD20 positive lymphomas cannot afford Rituximab. T-CHOP is a regimen that worth further investigation for this patient population.

Thus, this study has identified, for the first time, a safe and effective regimen for treating patients with DLBCL who cannot afford rituximab. In this study, no statistically significant difference in OS was found between the T-CHOP group and the CHOP group, which might be mainly attributable to the relatively small sample size of this study.

In conclusion, our study showed that T-CHOP was a highly effective and low-toxicity regimen that might be considered as a new regimen for the treatment of DLBCL patients who cannot afford rituximab. It is showed that the Bcl-2 positive/Bcl-6 negative DLBCL patients gained survival benefit from adding thalidomide to the CHOP regimen.

As a phase II clinical trial designed in 2005, there were some limitations of our study. First, the sample size is relatively small to detect the difference in survival. Second, we didn't introduce the GCB/non-GCB subtype detection in this study, and IHC detection of Bcl-2 and Bcl-6 was not done in every patient. Third, the dose-intensity of CHOP regimen in our study is lower than the standard regimen. Since there was no report about thalidomide combined with CHOP for newly diagnosed DLBCL patients published so far, our findings need to be further validated in the future.

## PATIENTS AND METHODS

### Patients

Patients age between 18 and 75 years old and had untreated diffuse large B cell lymphoma that had been diagnosed according to the World Health Organization classification. Additional enrollment criteria were as follows: an Eastern Cooperative Oncology Group performance status of 0-2; a more than 3 months life expectancy; and acceptable hematologic, hepatic, renal and cardiac function. Patients with a history of deep venous embolism (DVE) or pulmonary embolism (PE) were excluded. Pregnant and lactating women were also excluded.

### Ethical clearance

This trial was approved by the institutional review board, the Fudan University Shanghai Cancer Center Ethics Committee for Clinical Investigation on Dec 29th, 2005. All patients were told that they should take R-CHOP as the induction chemotherapy regimen if they can afford rituximab. Patients who cannot afford rituximab can choose to participate in this clinical trial. This trial is conducted in accordance with the Declaration of Helsinki and Good Clinical Practice. All patients provided written informed consent according to institutional guidelines.

### Randomization

Eligible patients were randomly assigned according to the sequence of the time they signed the informed consent form. The randomization table was generated by the Stata software.

### Treatment and assessment

Patients in the control group received CHOP regimen (cyclophosphamide 600mg/m^2^ d1, epirubicin 60mg/m^2^ d1, vincristine 1.4 mg/m^2^ [capped at 2.0 mg] d1, and prednisone 60mg/m^2^ per day on d1-5). Patients in the T-CHOP group also received thalidomide. Thalidomide 200 mg per day was administered on days 6-21 in the first cycle, if the patient can tolerate it well the dosage will be added to 400 mg per day on days 6-21 since the second cycle or he/she will continue to receive 200mg per day. Patients who had grade 4 neutropenia or febrile neutropenia after any cycle of chemotherapy were given G-CSF. The dose of cyclophosphamide and epirubicine will decrease 25% if the patient experience grade 4 hematological toxicity. CT imagings of lymphoma involved area were repeated every two cycles. No prophylactic anticoagulation medications were given to patients who received thalidomide. The treatment continued for a maximum of 8 cycles or until disease progression.

Tumor response and progression were determined according to the International Workshop Criteria [18]. The primary end point was CR rate, which include CR and CRu. The secondary end points include: ORR, PFS and OS. Adverse events were graded according to the National Cancer Institute Common Toxicity Criteria for Adverse Events (version 3.0) and reported in detail.

### Statistical analysis

Reported data showed that the CR rate in Chinese DLBCL patients treated with CHOP was 37.5-52.8% [20, 21]. In Gela 98-5 study, the CR rate is 37% and the CR+CRu rate is 63% for patients treated with CHOP [6]. We expected the CR rate of patients randomly assigned to receive CHOP or T-CHOP to be 40% and 70%, respectively. At least 31 patients are needed to be enrolled with 1:1 random assignment when significance was set at a one-sided 5% type I error and at least 80% power. Response rates in each treatment group were compared using chi-squared tests. Progression-free survival and overall survival were analyzed with log-rank tests and expressed as Kaplan-Meier plots or Life Tables plots. Pearson chi-squared test or Fisher's exact test was used to analyze categorical variables. P-values were one-tailed for comparing the response rate and two-tailed for other tests, and statistical significance was set at P < 0.05. Statistical analysis was performed with SPSS software version 16.0 (SPSS, Chicago, IL)

## SUPPLEMENTARY TABLES


