# Supplementary material for: Thalidomide enhanced the efficacy of CHOP chemotherapy in the treatment of diffuse large B cell lymphoma: A phase II study
Source: Oncotarget. 2016 Apr 25;7(22):33331–9. doi: 10.18632/oncotarget.8973 (PMC5078098; doi:10.18632/oncotarget.8973)
Supplement: Supplementary file 1 [file oncotarget-07-33331-s001.pdf]

SUPPLEMENTARY TABLES

Supplementary Table S1: Clinical characteristics in patients with positive Bcl-2 and positive Bcl-6 treated with T-CHOP or CHOP

Clinical variable	T- CHOP n(%)	CHOP n (%)	*P* value
Age			1.000*
≤60yr	5(62.5)	4(80)	
>60yr	3(37.5)	1(20)	
Stage			0.592*
I or II	5(62.5)	2(40)	
III or IV	3(37.5)	3(60)	
Serum lactate dehydrogenase			1.000*
≤250IU/L	5(62.5)	3(60)	
>250IU/L	3(37.5)	2(40)	
Performance status			0.385*
0 or 1	8(100)	4(80)	
2	0	1(20)	
Extranodal involvement			-
0 or 1	8(100)	5(100)	
≥2	0	0	
International prognostic index			1.000*
0 or 1	5(62.5)	3(60)	
2 or 3	3(37.5)	2(40)	
4 or 5	0	0	
Median cycles of chemo	6	8	0.293*

Abbreviations: T-CHOP, Thalidomide plus cyclophosphamide, doxorubicin, vincristine, and prednisone.
*Fisher's exact test

Supplementary Table S2: Clinical characteristics in patients with positive Bcl-2 and negative Bcl-6 treated with T-CHOP or CHOP

Clinical variable	T- CHOP n(%)	CHOP n (%)	*P* value
Age			1.000*
≤60yr	5(83.3)	5(100)	
>60yr	1(16.7)	0	
Stage			1.000*
I or II	3(50)	3(60)	
III or IV	3(50)	2(40)	
Serum lactate dehydrogenase			0.567*
≤250IU/L	4(66.7)	2(40)	
>250IU/L	2(33.3)	3(60)	
Performance status			0.455*
0 or 1	6(100)	4(80)	
2	0	1(20)	
Extranodal involvement			-
0 or 1	6(100)	5(100)	
≥2	0	0	
International prognostic index			0.242*
0 or 1	5(83.3)	2(40)	
2 or 3	1(17.7)	3(60)	
4 or 5	0	0	
Median cycles of chemo	6	4	0.175*

Abbreviations: T-CHOP, Thalidomide plus cyclophosphamide, doxorubicin, vincristine, and prednisone.
*Fisher's exact test

Supplementary Table S3: Clinical characteristics in patients with negative Bcl-2 and positive Bcl-6 treated with T-CHOP or CHOP

Clinical variable	T- CHOP n(%)	CHOP n (%)	*P* value
Age			0.333*
≤60yr	0	2(100)	
>60yr	2(100)	0	
Stage			1.000*
I or II	1(50)	2(100)	
III or IV	1(50)	0	
Serum lactate dehydrogenase			0.333*
≤250IU/L	0	2(100)	
>250IU/L	2(100)	0	
Performance status			-
0 or 1	2(100)	2(100)	
2	0	0	
Extranodal involvement			-
0 or 1	2(100)	2(100)	
≥2	0	0	
International prognostic index			0.333*
0 or 1	0	2(100)	
2 or 3	2(100)	0	
4 or 5	0	0	
Median cycles of chemo	6	6	1.000*

Abbreviations: T-CHOP, Thalidomide plus cyclophosphamide, doxorubicin, vincristine, and prednisone.
*Fisher's exact test

Supplementary Table S4: Clinical characteristics in patients with negative Bcl-2 and negative Bcl-6 treated with T-CHOP or CHOP

Clinical variable	T- CHOP n(%)	CHOP n (%)	*P* value
Age			1.000*
≤60yr	1(100)	3(75)	
>60yr	0	1(25)	
Stage			-
I or II	1(100)	4(100)	
III or IV	0	0	
Serum lactate dehydrogenase			0.400*
≤250IU/L	0	3(75)	
>250IU/L	1(100)	1(25)	
Performance status			-
0 or 1	1(100)	4(100)	
2	0	0	
Extranodal involvement			-
0 or 1	1(100)	4(100)	
≥2	0	0	
International prognostic index			1.000*
0 or 1	1(100)	3(75)	
2 or 3	0(0)	1(25)	
4 or 5	0	0	
Median cycles of chemo	6	6	-

Abbreviations: T-CHOP, Thalidomide plus cyclophosphamide, doxorubicin, vincristine, and prednisone.
*Fisher's exact test
